# Agenesis of the gall bladder, an unexpected finding during laparoscopy; case report

**DOI:** 10.1016/j.amsu.2020.04.043

**Published:** 2020-05-14

**Authors:** Sardar Hassan Arif, Ayad Ahmad Mohammed

**Affiliations:** Department of Surgery, College of Medicine, University of Duhok, Kurdistan Region, Iraq

**Keywords:** Gall bladder agenesis, Cholecystitis, MRCP, Diagnostic laparoscopy

## Abstract

Congenital agenesis of the gall bladder is a very rare ranging from 0.02% to 0.002% in clinical practice. There is complete absence of the gall bladder with normal intra and extra hepatic biliary tree. The exact etiology remains unclear. Low index of suspicion and failure of routine investigations will result in its unexpected discovery during surgery. The condition usually results in diagnostic dilemma both before surgery and intraoperatively.

A 25-year-old lady presented with repeated attacks of right side abdominal pain for 1 year. Abdominal examination showed tenderness on palpation in the right hypochonrdium. Abdominal ultrasound showed normal common bile duct with suspicion of small contacted gall bladder. MRCP showed extrahepatic biliary tree and not visualized gall bladder. During diagnostic laparoscopy exploration of the whole peritoneal cavity was performed. The gall bladder was not visualized after complete visualization of biliary anatomy. The appendix was inflamed with multiple adhesions with the bowel. The cecum was high placed in the sub-hepatic region. Laparoscopic appendicectomy was performed.

Patients with gall bladder agenesis surprisingly have symptoms similar to cholecystitis, the pain may be attributed to cholangitis, biliary stones, or sphincter of Oddi dysfunction. When the condition diagnosed at operation extensive dissection to identify the gall bladder must be avoided because it may result in biliary injury.

## Highlights

• Congenital agenesis of the gall bladder is a very rare.
• Many patients surprisingly have symptoms similar to cholecystitis.
• Extensive dissection to identify the gall bladder must be avoided because it may result in biliary injury.

## 1 Introduction

Congenital agenesis of the gall bladder is a very rare anatomical abnormality of the biliary tree in which there is complete absence of the gall bladder with normal intra and extra hepatic biliary tree. Most cases are associated with hypoplastic or agenesis of the cystic duct. The exact incidence is not clear, but it may range from 0.03%–0.07% from autopsy samples, its incidence in most of the clinical series is ranging from 0.02% to 0.002%. This anomaly was first described in by Lemery and Bergman 1701 and 1702, since that some cases are reported worldwide [[1], [2], [3], [4], [5]].

During embryonal life, the gall bladder arises as a primitive bud from the hepatic diverticulum which is derived from the primitive foregut, failure of further development will result in complete agenesis of the gall bladder and the cystic duct [6].

The exact etiology of this anomaly remains unclear, although this condition has been reported in some families suggesting a genetic base. Low index of suspicion and failure of routine investigations to discover this anomaly, will result in its unexpected discovery during surgery, or many cases are discovered at autopsy samples [1,6].

Most affected individuals are asymptomatic, some patients have right upper quadrant abdominal pain suggesting gall bladder disease, and some have repeated attacks of cholangitis and jaundice, nausea, or intolerance to fatty foods [6,7].

In most cases ultrasound examination fails to diagnose the condition however MRCP or ERCP if performed will show the anatomy and fail to visualize the gall bladder in most of the cases. The condition usually results in diagnostic dilemma both before surgery and intraoperatively [2,7].

The work of this case report has been reported in line with the SCARE criteria [8].

## 2 Patient information

**Clinical findings:** A 25-year-old lady presented with chronic right side abdominal pain for 1 year, the pain was dull aching in nature, relieved by analgesics and associated with nausea and no vomiting. The patient has repeated attacks of the same pain with variable intensities.

The patient has negative medical and surgical histories, and she had no history of chronic drug usage. She had repeated visits to the hospital for the same pain.

During examination she had normal vital signs, with no fever. The general examination was unremarkable. Abdominal examination showed tenderness on palpation in the right hypochondrial region.

**Diagnostic assessment:** The complete blood picture showed mildly elevated white blood cell count (11.9 mm^3^), with normal hemoglobin and platelet count.

**Fig. 1:**
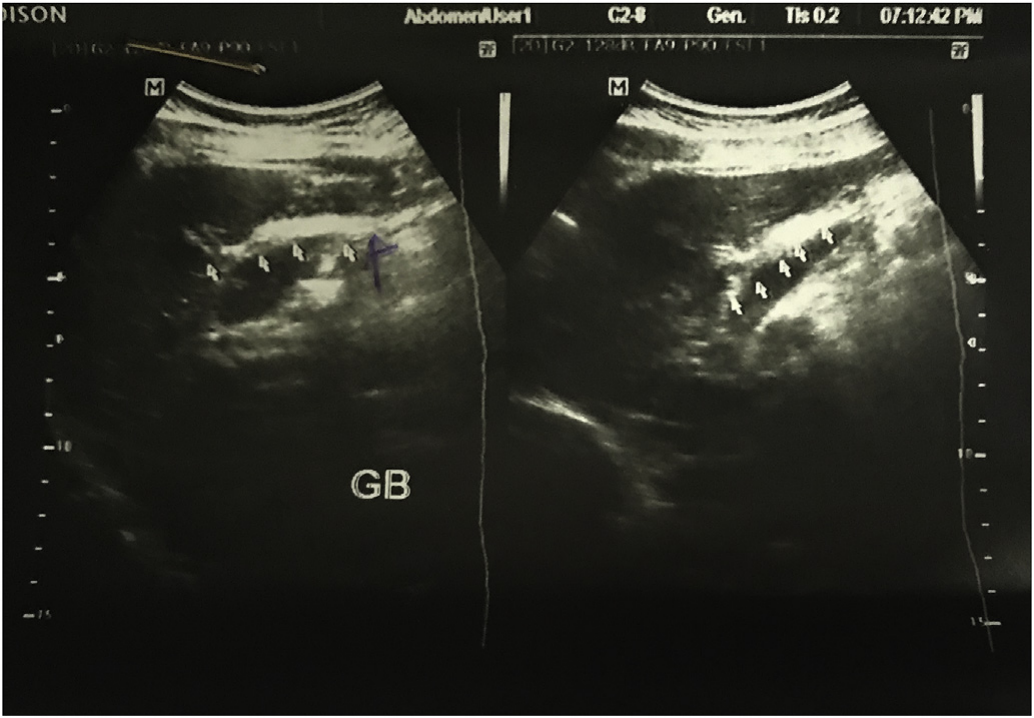
An ultrasound picture of the biliary tree showing a normal caliber of the common bile duct with suspicion of small and contracted gall bladder (the white arrows).

Abdominal ultrasound showed normal liver and spleen. The common bile duct was normal, with suspicion of small contacted gall bladder. Other intra-abdominal and pelvic organs were normal. Fig. 1.

**Fig. 2:**
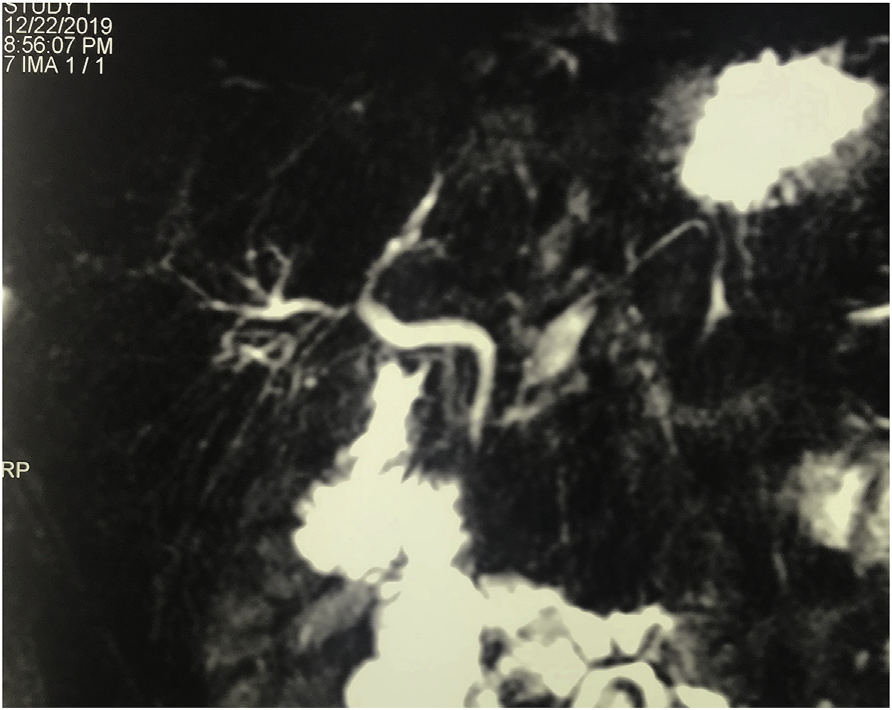
MRCP picture showing normal caliber of the common bile duct with normal both right and left hepatic duct, the gall bladder is not visualized.

**Fig. 3:**
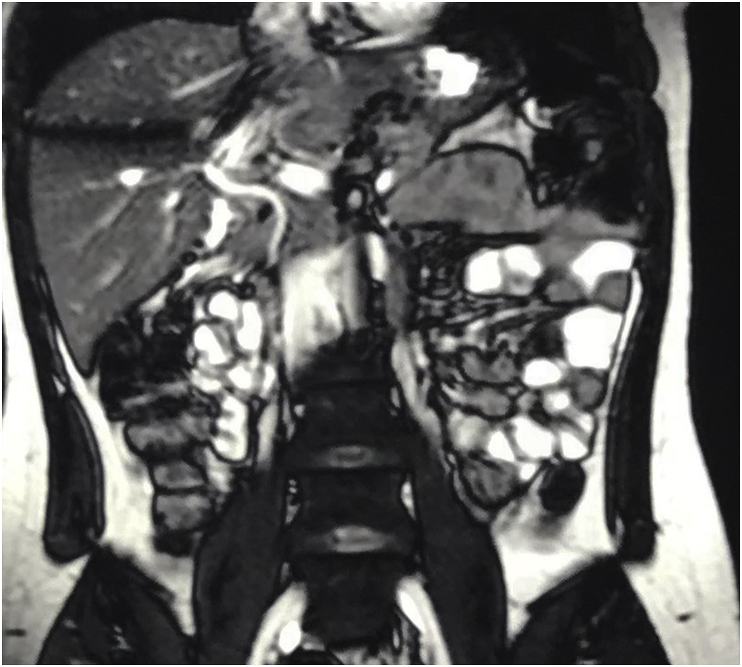
MRCP picture showing normal caliber of the common bile duct with normal both right and left hepatic duct, the gall bladder is not visualized.

Magnetic resonance cholangiopancreatography (MRCP) showed normal caliber common bile duct (4mm) in diameter, with normal both right and left hepatic duct, the gall bladder was not visualized. The pancreatic duct was normal in size (2mm). Fig. 2, Fig. 3.

The patient received oral analgesics with little response, and had repeated admissions due to similar pain.

**Fig. 4:**
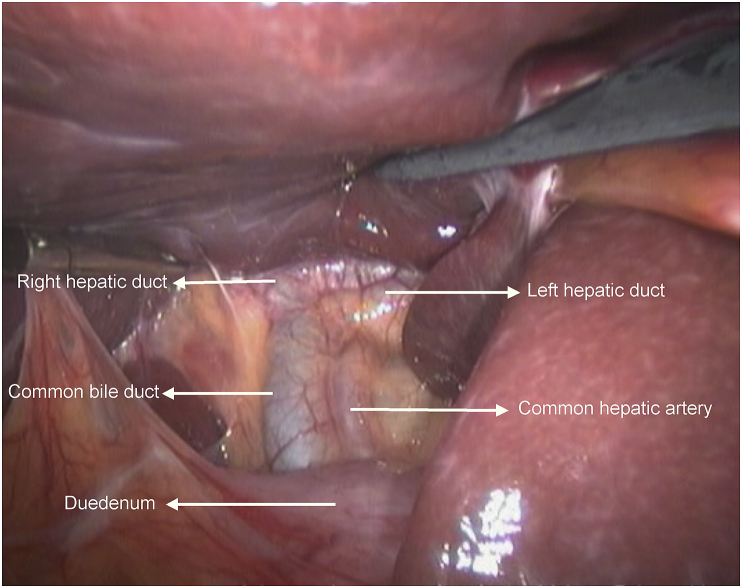
A laparoscopic view of the porta hepatic and sub-hepatic region showing the right and left hepatic duct, the common bile duct, the common hepatic artery, and the duodenum (arrowed).

**Fig. 5:**
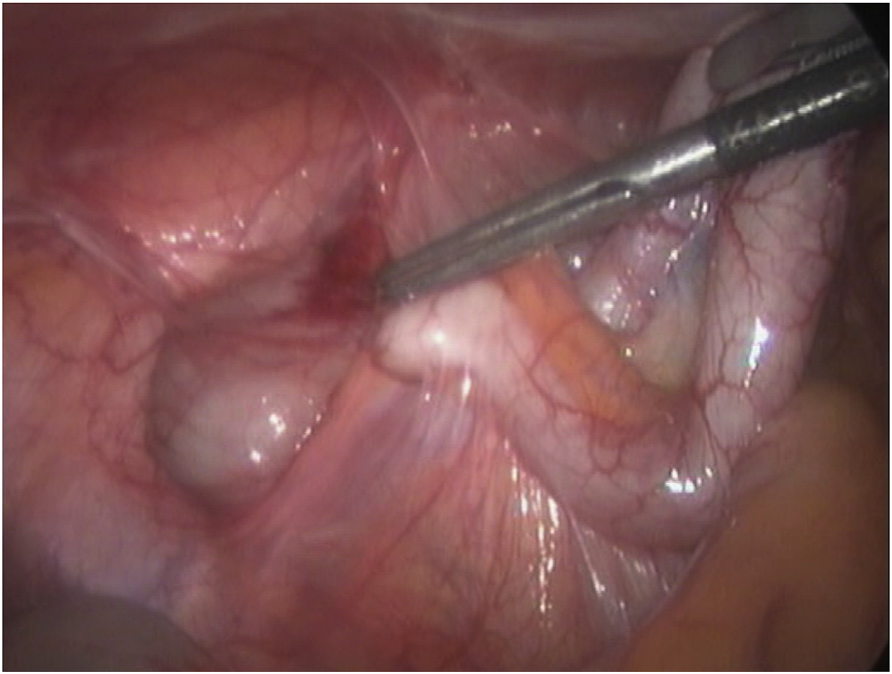
A laparoscopic picture showing the inflamed appendix with adhesions with the surrounding organs suggesting previous attacks of inflammation.

**Therapeutic Intervention:** Decision for diagnostic laparoscopy was done. During laparoscopy exploration of the whole peritoneal cavity was performed. The gall bladder was not visualized after complete visualization of biliary anatomy. The appendix was inflamed with multiple adhesions with the bowel. The cecum was high placed in the sub-hepatic region. Laparoscopic appendicectomy was performed. Fig. 4, Fig. 5.

**Follow-up and outcomes:** The patient was admitted for 2 days after surgery with no postoperative complications. Follow up was done for 6 months after surgery with improvement of the general condition and no similar attacks of the pain.

## 3 Discussion

Anomalies of the biliary tree are numerous ranging from anomalies of the shape of the gall bladder, location, number, or complete absence or agenesis [9,10].

Retrospective data showed that the presented case is the only case of gall bladder agenesis reported from approximately 11750 cases of laparoscopic cholecystectomies which were performed in this center for the last 15 years, although some other anomalies were reported such as ectopic, midline and duplicated gallbladders.

It has been reported that this agenesis of the gall bladder may be associated with some other anomalies of the cardiovascular system, hepato-biliary tree, the genitourinary system, and gastrointestinal system [6,11].

Bennion divided this condition into 3 categories based on the clinical situation; healthy individuals with no clinical symptoms, symptomatic patients and those who have associated congenital anomalies [3].

Symptomatic patients often are diagnosed as having cholecystitis and surprisingly some patients will be diagnosed as gall bladder disease and undergo surgery when the condition will be diagnosed at operation. The pain in patients with gall bladder agenesis, may be attributed to cholangitis, biliary stones, or sphincter of Oddi dysfunction [4,6].

In this case there were no any signs of inflammation around biliary tree but we found abnormal high appendix with pictures of inflammation.

New diagnostic techniques such as MRCP and biliary scintigraphy can potentially detect biliary anomalies HIDA (Hepatobiliary iminodiacetic acid) scan may be performed in some with gall bladder symptoms, in those patients non-visualization of the gall bladder may be attributed to cystic duct obstruction, so it may not be as informative as MRCP [3,5].

When the condition is diagnosed preoperatively and there are no biliary stones with MRCP, ERCP must be performed to detect any small biliary stones or biliary sludge. Some patients improved clinically after the conservative approach, other may improve after ERCP and sphincterotomy [3,6].

Patients with stones in the common bile duct must undergo stone extraction of the stones, however when the symptoms are atypical, diagnostic laparoscopy may be warranted [3].

Some authors reported dramatic improvement of the symptoms after exploratory surgery.

In this case we didn't dissect at the site of gall bladder because it was very clear there was absent gall bladder and dissection at the region of the extrahepatic biliary tree to identify the gall bladder may result in biliary injury [1,3].

Agenesis of the gall bladder is a very rare finding during clinical practice. Patients are usually asymptomatic. Ultrasound usually fail to diagnose this condition. MRCP can diagnose this anomaly and delineate the anatomy of the biliary tree. Extensive dissection identify the gall bladder may result in biliary injury and to consult more expert surgeon before any interference.

## Ethical approval

No ethical committee approval was needed; consent have been taken from the patient to report the finding in this case report.

## Funding source

No source of funding other than the authors.

## Author contribution

The surgeon who performed the procedure: Dr Sardar Hassan Arif and Dr Ayad Ahmad Mohammed Study design, writing, and the final approval of the manuscript: Dr Ayad Ahmad Mohammed and Dr Sardar Hassan Arif.

## Registration of research studies

N/A.

## Guarantor

Dr Ayad Ahmad Mohammed.

## Patient perspective

I had repeated attacks of abdominal pain and I thought it was due to gall stones, after surgery I felt too much better and I have been told that no gall bladder was found during surgery.

## Informed consent

An informed written consent was taken from the patient for reporting this case and the accompanying images.

## Provenance and peer review

Not commissioned, externally peer reviewed.

## Declaration of competing interest

No conflicts of interest present.
